# X-Band Radar Cross-Section of Tandem Helicopter Based on Dynamic Analysis Approach

**DOI:** 10.3390/s21010271

**Published:** 2021-01-03

**Authors:** Zeyang Zhou, Jun Huang

**Affiliations:** School of Aeronautic Science and Engineering, Beihang University, Beijing 100191, China; junh@china.com

**Keywords:** a double main rotor, helicopter, tandem layout, electromagnetic scattering characteristics

## Abstract

In order to study the radar signature of a tandem helicopter in the X-band, a dynamic analysis approach (DAA) is presented to determine its radar cross-section (RCS) under different influence factors. The basic passage time, rotation speed, observation angle, rotor disk inclination, fuselage attitude angle and Doppler feature are studied and discussed in detail. The results show that the dynamic characteristics of the rotor RCS will bring significant changes to the peak and average values of the helicopter RCS. Within a given observation angle range, a larger elevation angle is undesirable for helicopter stealth. The inclination of the rotor disc will affect the many small peaks and local fluctuations of the helicopter RCS. The positively increased attitude angle will have an undesirable effect on the average RCS and dynamic characteristics of the helicopter. The DAA is feasible and effective for studying the radar cross-section of a tandem helicopter.

## 1. Introduction

As a uniquely configured aircraft, the tandem helicopter has the advantages of heavy load, small space size and high hovering efficiency, which makes it widely used in transportation, passenger transportation and rescue tasks. Hence, studying the electromagnetic scattering characteristics of tandem helicopters has important academic significance and engineering value [1,2,3].

For the solution of the radar cross-section (RCS) of aircraft targets, many research methods and work have been presented, including the method of moment (MOM) [1], physical optics (PO) [4,5], flight testing [6,7] and physical theory of diffraction (PTD) [8]. The combination of the fact-based asymptotic method and the geometrical optics (GO) and PO hybrid method is used to calculate the RCS of a large target [9]. The quasi-static principle (QSP) is used to simulate the motion of the blade, and the RCS of the rotor is obtained by adopting PO and equivalent currents method [10]. Different from a single rotor structure, a tandem helicopter usually uses a dual rotor configuration, which brings a new source of scattering to the aircraft [11,12,13]. It can be seen that these existing RCS calculation methods can solve the electromagnetic scattering characteristics of the static and a small amount of quasi-static rotors. This obviously cannot satisfy the determination of the radar characteristics of the two large main rotors of the tandem helicopter [14].

In order to analyze the dynamic RCS response of a compound helicopter, a multi-rotor, dynamic scattering calculation method is presented [15]. This processing approach can bring support and reference for the solution of the radar characteristics of the tandem helicopter [16,17]. A dynamic transformation method based on PO and PTD was established to calculate the electromagnetic scattering characteristics of coaxial helicopters [18]. When controlling the flight attitude, the tilt angle of the rotor disc will also change the RCS of the helicopter [15,19,20]. The difference in structure from conventional unmanned helicopters is that unmanned tandem helicopters have no slender tail boom, with two rotors of the same size arranged in the longitudinal direction, one in the front and one in the back, and the rotors have the same rotation speed and opposite rotation directions [21,22,23]. In order to ensure the accuracy of simultaneous localization and mapping (SLAM) in the millimeter-wave band [24,25], a millimeter-wave radar SLAM assisted by the RCS feature of the target and inertial measurement unit is presented. With the extensive equipment of dual-band radars on various weapon platforms, the study of the electromagnetic scattering characteristics of helicopters in the X-band has also become important.

Previous studies on helicopter radar cross-sections were mostly static electromagnetic scattering calculations. In the past two years, dynamic RCS response analysis of rotor, coaxial helicopter and the compound helicopter has gradually appeared. Unlike the conventional layout of a helicopter, the tandem helicopter has two large main rotors, which are installed on the lower tower at the nose and the higher tower at the tail, noting that this design allows the two main rotors to affect the aircraft’s RCS within the circumferential azimuth range. According to the core idea of the dynamic scattering method [3], we apply this approach to the tandem helicopter and expand the independent calculation of the influence of the rotor disk tilt and the analysis of the Doppler characteristics. Therefore, this paper attempts to present a dynamic analysis approach (DAA) to determine the RCS of a tandem helicopter. In view of the uniqueness of this aircraft, important influencing factors will be analyzed and discussed, including rotation speed, observation direction, rotor disc inclination and attitude angle. It can be seen that the research on the RCS of the tandem helicopter has important engineering application value and academic significance for the stealth design of the helicopter.

In this article, the DAA is presented in Section 2. Models of helicopter and rotor are given in Section 3. The results of dynamic electromagnetic scattering are discussed in Section 4. Finally, the full text is summarized.

## 2. Dynamic Analysis Approach

**Figure 1:**
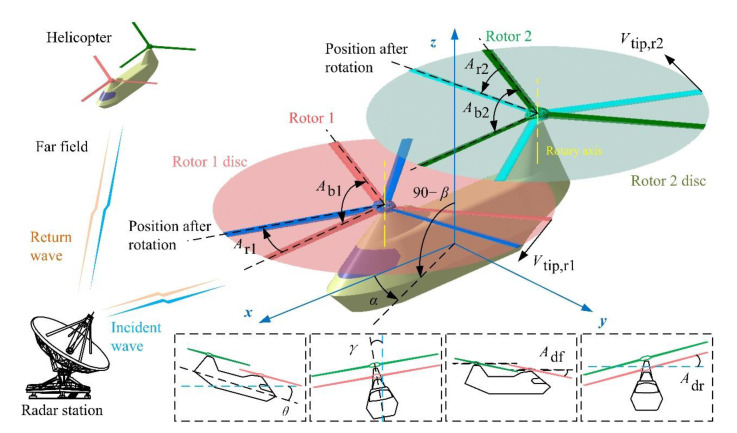
Schematic diagram of dynamic electromagnetic scattering of the tandem helicopter.

The schematic of dynamic scattering of a tandem helicopter is shown in Figure 1, where *A*_b_ represents the angle between adjacent blades of the rotor, and the subscript numbers (1 and 2) refer to the serial number of the corresponding rotor, *A*_r_ is the rotation angle of the rotor, *α* is the azimuth of the radar station, *β* is the elevation angle, *V*_tip_ is the velocity of the blade tip, *θ* is the pitch angle of the helicopter, *γ* is the roll angle of the helicopter, *A*_df_ is the forward tilt angle of the rotor disc, *A*_dr_ is the right tilt angle of the rotor disc. When the inclination angle of the rotor disc is equal to 0, the rotation axes of the two rotors are parallel to the *z*-axis.

### 2.1. Dynamic Process Simulation

The helicopter consists of two main rotors and a fuselage; thus, its initial model can be expressed as follows:

(1) $$m_{h}\left(t=\;0\right)=\left\{m_{r1}\left(t=\;0\right),m_{r2}\left(t=\;0\right),m_{f}\left(t=\;0\right)\right\}$$

where *t* is time, *m*_h_ is the model of the tandem helicopter, *m*_r1_ is the model of rotor 1, *m*_r2_ is the model of rotor 2, *m*_f_ is the model of the fuselage [3,15]. In the current coordinate system, when rotor 1 rotates, its dynamic model can be updated to:

(2) $$M_{r1}^{x}\left(m_{r1}\left(t=0\right)\right)=M_{r1}\left(x\left(m_{r1}\left(t=0\right)\right)-X_{r1}\right)|\gamma =\theta =0\begin{array}{cc} , & A_{\mathrm{df}}=A_{\mathrm{dr}}=0 \end{array}$$

(3) $$M_{r1}^{xz}\left(m_{r1}\left(t\right)\right)=\left[\begin{array}{ccc} \cos A_{r1}\left(t\right) & -\sin A_{r1}\left(t\right) & 0\\ \sin A_{r1}\left(t\right) & \cos A_{r1}\left(t\right) & 0\\ 0 & 0 & 1 \end{array}\right]\cdot M_{r1}^{x}\left(m_{r1}\left(t=0\right)\right)|\begin{array}{l} \begin{array}{c} \gamma =\theta =0 \end{array}\\ A_{\mathrm{df}}=A_{\mathrm{dr}}=0 \end{array}$$

where ***M***_r1_ is the grid coordinate matrix of rotor 1, superscript *x* represents the translation along the *x*-axis, superscript *xz* represents the rotation around the *z*-axis in the current operation, *X*_r1_ is the distance between the axis of rotor 1 and the *z*-axis in the initial state. When the rotor disc is tilted forward, the dynamic model of rotor 1 can be updated to:

(4) $$M_{r1}^{y}\left(m_{r1}\left(t\right)\right)=\left[\begin{array}{ccc} \cos A_{\mathrm{df}}\left(t\right) & 0 & -\sin A_{\mathrm{df}}\left(t\right)\\ 0 & 1 & 0\\ \sin A_{\mathrm{df}}\left(t\right) & 0 & \cos A_{\mathrm{df}}\left(t\right) \end{array}\right]\cdot M_{r1}^{xz}\left(m_{r1}\left(t=0\right)\right)|\begin{array}{l} \begin{array}{c} A_{\mathrm{dr}}=0 \end{array}\\ \gamma =\theta =0 \end{array}$$

where the superscript *y* represents the rotation around the *y*-axis. When the paddle is tilted to the right, the dynamic model of rotor 1 can be expressed as:

(5) $$M_{r1}^{xy}\left(m_{r1}\left(t\right)\right)=\left[\begin{array}{ccc} 1 & 0 & 0\\ 0 & \cos A_{\mathrm{dr}}\left(t\right) & -\sin A_{\mathrm{dr}}\left(t\right)\\ 0 & \sin A_{\mathrm{dr}}\left(t\right) & \cos A_{\mathrm{dr}}\left(t\right) \end{array}\right]\cdot M_{r1}^{y}\left(m_{r1}\left(t=0\right)\right)|\begin{array}{l} \gamma =\theta =0 \end{array}$$

where the superscript *xy* represents the rotation around the *x*-axis in the current operation. For the rotational movement of rotor 2, there are the following descriptions:

(6) $$M_{r2}^{x}\left(m_{r2}\left(t=0\right)\right)=M_{r2}\left(x\left(m_{r2}\left(t=0\right)\right)+X_{r2}\right)|\gamma =\theta =0\begin{array}{cc} , & A_{\mathrm{df}}=A_{\mathrm{dr}}=0 \end{array}$$

(7) $$M_{r2}^{xz}\left(m_{r2}\left(t\right)\right)=\left[\begin{array}{ccc} \cos A_{r2}\left(t\right) & -\sin A_{r2}\left(t\right) & 0\\ \sin A_{r2}\left(t\right) & \cos A_{r2}\left(t\right) & 0\\ 0 & 0 & 1 \end{array}\right]\cdot M_{r2}^{x}\left(m_{r2}\left(t=0\right)\right)|\begin{array}{l} \begin{array}{c} \gamma =\theta =0 \end{array}\\ A_{\mathrm{df}}=A_{\mathrm{dr}}=0 \end{array}$$

where ***M***_r2_ is the grid coordinate matrix of rotor 2, superscript *x* represents the translation along the *x*-axis, superscript *xz* represents the rotation around the *z*-axis in the current operation, *X*_r2_ is the distance between the axis of rotor 2 and the *z*-axis in the initial state, noting that the update of the rotor 2 model caused by the disc tilt is similar to the operation of rotor 1. Return the rotor model matrix obtained above to their initial position, and merge with the model of the fuselage, the dynamic model of the helicopter can be obtained as:

(8) $$M_{h}^{}\left(m_{h}\left(t\right)\right)=\left[\begin{array}{ccc} M_{r1}^{z}\left(m_{r1}\left(t\right)\right), & M_{r2}^{z}\left(m_{r2}\left(t\right)\right), & M_{f}^{}\left(m_{f}\left(t=\;0\right)\right) \end{array}\right]|\begin{array}{l} \gamma =\theta =0 \end{array}$$

where the superscript z represents the transformation about the *z*-axis. When the attitude angle of the fuselage changes, the helicopter model can be updated as follows:

(9) $$M_{h}^{x}\left(m_{h}\left(t\right)\right)=\left[\begin{array}{ccc} 1 & 0 & 0\\ 0 & \cos\gamma & -\sin\gamma \\ 0 & \sin\gamma & \cos\gamma \end{array}\right]\cdot M_{h}^{}\left(m_{h}\left(t\right)\right)|\begin{array}{l} \theta =0 \end{array}$$

(10) $$M_{h}^{y}\left(m_{h}\left(t\right)\right)=\left[\begin{array}{ccc} \cos\theta & 0 & -\sin\theta \\ 0 & 1 & 0\\ \sin\theta & 0 & \cos\theta \end{array}\right]\cdot M_{h}^{x}\left(m_{h}\left(t\right)\right)$$

where superscript *x* represents the rotation around the *x*-axis in the current operation, superscript *y* represents the rotation around the *y*-axis in the current operation. When incident radar waves illuminate the helicopter, the illuminated area on its surface can be extracted as:

(11) $$S\left(t\right)⇐M_{h}^{y}\left(m_{h}\left(t\right)\right)$$

where *S*(*t*) is the illuminated area of the helicopter.

### 2.2. Electromagnetic Scattering Calculation

In the PO method, the far-field electric field formula can be expressed as follows:

(12) $$E_{s}^{\left(m_{h}\left(t\right)\right)}=\frac{e^{-jkr}}{4\pi r}\left(-j\omega \mu \right)\int_{S\left(t\right)}J_{s}e^{-jks\cdot r^{\prime }\left(t\right)}dS+jks\frac{1}{\varepsilon }\int_{S\left(t\right)}\rho_{s}e^{-jks\cdot r^{\prime }\left(t\right)}dS$$

where *k* is the wavenumber, *ω* is the angular frequency, *μ* is the magnetic permeability, ***J***_s_ is the surface current, ***s*** means radiation direction, ***r***′ is the source point coordinate vector, *r* is the field point, ε is the dielectric constant, *ρ*_s_ is the charge density, d*S* is the integral facet [8,18]. Under the irradiation of plane waves, the target RCS calculation formula can be written as:

(13) $$\sqrt{\sigma_{F}\left(t\right)}=\underset{R\to \infty }{\lim}\sqrt{4\pi R^{2}}\frac{e_{s}\cdot E_{s}^{\left(m_{h}\left(t\right)\right)}}{\left|E_{i}\right|}$$

where *R* is the distance between the field point and the source point, ***e***_s_ is the direction of the scattered electric field, *E*_i_ is the electric field intensity at the point of incidence.

For the far-field, the RCS formula can be further transformed into:

(14) $$\sqrt{\sigma_{F}\left(t\right)}=\sqrt{4\pi R^{2}}\frac{1}{\left|E_{i}\right|}\left(-j\omega \mu \frac{e^{-jkr}}{4\pi r}\int_{S\left(t\right)}e_{s}\cdot J_{s}e^{-jks\cdot r^{\prime }\left(t\right)}dS\right)$$

Note that the surface current could be expressed as:

(15) $$J_{s}=2n\;\times H_{i}$$

where ***n*** is the normal vector outside the surface element, ***H***_i_ is the magnetic field intensity of the incident point. Considering the relationship between electric field and magnetic field, the calculation formula of RCS can be obtained as:

(16) $$\sqrt{\sigma_{F}\left(t\right)}=\frac{jk}{\sqrt{\pi }}n\cdot \left(e_{s}\times h_{i}\right)e^{jkw\cdot r_{0}\left(t\right)}I\left(t\right)$$

(17) w=s−i

where ***h***_i_ is the direction of the incident magnetic field, ***r***_0_ represents the coordinate vector of the reference point on the integral surface element, ***i*** is the unit vector of the incident wave, *I*(*t*) is an integral expression, which can be obtained according to the following calculation when triangular facets are used:

(18) $$I\left(t\right)=\{\begin{array}{ll} \frac{e^{jkw\cdot r_{m}\left(t\right)}}{jk{\left|p\right|}^{2}}\sum_{m=1}^{3}p\cdot L_{m}sinc\left(\frac{kw\cdot L_{m}}{2}\right) & \begin{array}{cc} , & \left|p\right|\neq 0 \end{array}\\ A_{f} & \begin{array}{cc} , & \left|p\right|=0 \end{array} \end{array}$$

(19) sinc(x)=sinx/x

where ***L***_m_ is the vector of the *m*-th edge on the facet, *A*_f_ is the area of the integral facet; for more details, please refer to the literature [5,26], noting that ***p*** is a defined vector cross product:

(20) p=n×w

PTD is used to solve the edge diffraction contribution of the target; for more information about PTD, please refer to the literature [5,8]. Therefore, the total RCS can be written as:

(21) $$\sigma \left(t\right)={\left|\sum_{i=1}^{N_{F}(t)}\left(\sqrt{\sigma_{F}(t)}\right)\begin{array}{c} i \end{array}+\sum_{j=1}^{N_{E}(t)}\left(\sqrt{\sigma_{E}(t)}\right)\begin{array}{c} j \end{array}\right|}^{2},\begin{array}{cc} & t\in \left[0,T_{\mathrm{obs}}\right] \end{array}$$

where *σ* is the radar cross-section, subscript E represents the edge contribution, and F represents the facet contribution. *N*_E_ is the number of edges, *N*_F_ is the number of facets, and *T*_obs_ is the recommended length of time for observation:

(22) $$T_{\mathrm{obs}}\geq \max\left\{t_{b,r1},t_{b,r2}\right\}|A_{\mathrm{df}}=A_{\mathrm{dr}}=0,\begin{array}{cc} & \gamma =\theta =0 \end{array}$$

(23) $$t_{b}=\frac{A_{b}}{\omega_{r}}\cdot \frac{\pi }{180}\begin{array}{cc} , & A_{b}=\frac{360}{N_{b}} \end{array}$$

where *t*_b_ is basic passage time, equal to the time it takes for the blade to rotate through the angle between two adjacent blades, *N*_b_ is the number of blades of the rotor, *ω*_r_ is the angular velocity of the rotor.

(24) $$T_{\mathrm{obs}}\geq \max\left\{t_{b,\max},N_{b,r1}\cdot t_{b,r1},N_{b,r2}\cdot t_{b,r2}\right\}|\forall p_{a}\in \left\{\begin{array}{cccc} A_{\mathrm{df}}, & A_{\mathrm{dr}}, & \gamma , & \theta \end{array}\right\},\begin{array}{cc} & p_{a}\neq 0 \end{array}$$

(25) $$t_{b,\max}=\max\left\{t_{b,r1},t_{b,r2}\right\}$$

where *p*_a_ is a custom angle parameter. The existence of *T*_obs_ can better present the complete dynamic characteristics of the RCS of the rotor or helicopter.

### 2.3. Doppler Analysis

Translate the current coordinate system to the center of rotor 1, the distance between any point *P* on the rotor surface and the radar can be expressed as:

(26) $$R_{P}\left(t\right)=\sqrt{{\left(R_{0}\right)}^{2}+{\left(l_{P}\left(x,y\right)\right)}^{2}+2l_{P}\left(x,y\right)R_{0}\cos\beta_{0}\cos\left(\omega_{r1}t+\varphi_{0}+\varphi \left(x,y\right)\right)}$$

where *R*_0_ is the distance between the radar and the rotation center of the rotor, *l_P_* is the distance from point *P* to the center of the rotor, *β*_0_ is the elevation angle of the radar beam, *φ* is the rotor phase, subscript 0 represents the initial phase:

(27) $$l_{P}\left(x,y\right)=\sqrt{x^{2}+y^{2}}$$

(28) $$\varphi \left(x,y\right)=\arctan\frac{y}{x}$$

(29) $$\beta_{0}=A_{\mathrm{df}}+\beta$$

where *A*_dr_ = 0. The angle between the radar line of sight and the vertical direction of point *P* at any time can be calculated as:

(30) $$\theta =\arccos\frac{R_{0}\sin\beta_{0}}{R_{P}\left(t\right)}$$

When the main body of the rotor translates along the *x*-direction at speed *V*_m_, the laser echo at point *P* can be expressed as:

(31) $$S_{P}\left(t\right)=\exp\left\{j\frac{4\pi \cos\beta_{0}}{\lambda }\left[l_{P}\cos\left(\omega_{r1}t+\varphi_{0}\right)+V_{m}t\right]\right\}$$

where *λ* is the wavelength. Then the micro-Doppler frequency at point *P* is:

(32) $$f_{MD}=\frac{2V_{m}\cos\beta_{0}}{\lambda }+\frac{4\pi n_{r1}l_{P}}{\lambda }\sin\left(2\pi n_{r1}t+\varphi_{0}\right)$$

where *n*_r1_ is the rotating speed of rotor 1. The Doppler frequency of the blade tip is usually the most obvious; thus, it can be extracted as:

(33) $$F_{\mathrm{dt}}=f_{MD}|\max(l_{P}\left(x,y\right))$$

Regardless of translational motion, it can be seen from the literature [10] that the maximum Doppler frequency caused by the rotation of the rotor can be obtained when electromagnetic waves are incident parallel to the plane of the rotor disk:

(34) $$f_{\mathrm{dmax}}=\frac{2f\omega_{r1}R_{r1}}{c}$$

where *f* represents the frequency of the radar wave, *ω*_r1_ represents the angular velocity of rotor 1, *R*_r1_ is the radius of rotor 1. The normalized processing of the rotor radar cross-section can be expressed as:

(35) $$\sigma_{n}\left(t\right)=\frac{\sigma \left(t\right)}{\max\left(\sigma \left(t\right)\right)-\min\left(\sigma \left(t\right)\right)}$$

In fact, *f*_dmax_ is slightly smaller than the maximum of *F*_dt_ because the real rotor has a width, that is, the chord length, which leads to the maximum Doppler frequency appearing at the tip of the leading or trailing edge of the rotor.

### 2.4. Method Verification

**Figure 2:**
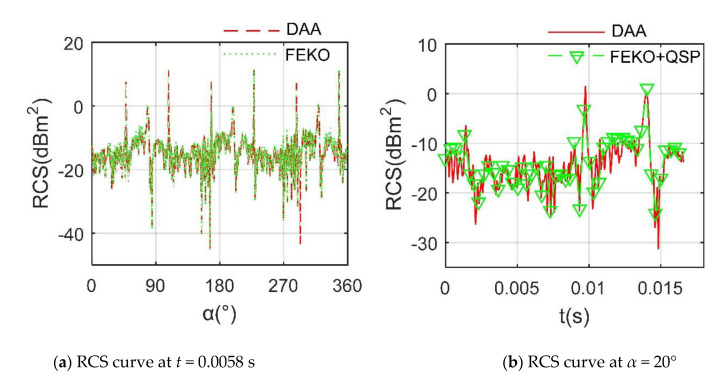
The validation of the radar cross-section (RCS) algorithm on rotor 1, *f*_RH_ = 10 GHz, *β* = 10°.

The validation of DAA is presented in Figure 2, where *f*_RH_ refers to radar wave frequency and horizontal polarization, rotating speed of rotor 1 *n*_r1_ = 1200 r/min. The PO + method of the moment (MOM)/multilevel fast multipole method (MLFMM) in FEKO is used to calculate the RCS of the target and compare it with the result of DAA [3]. For the case of *t* = 0.0058 s, it can be seen that the two RCS curves are generally similar, including peak size, peak position and overall trend. In the range of 56.5°~72.75° and 325.3°~347° azimuth angles, the calculation result of FEKO is slightly higher than that of DAA, where the RCS means of the former is −9.36 dBm^2^, while that of the latter is −9.61 dBm^2^. These results show that DAA is accurate to calculate the transient or static RCS of the target.

For the RCS curve at *α* = 20°, the quasi-static principle (QSP) is used to discretize the rotational motion of the rotor so that the conventional method can proceed to the calculation of rotor dynamic RCS. It can be seen that the RCS curve determined by DAA can help better pass through the discrete data points calculated by FEKO, but DAA clearly reflects the subtle changes in rotor RCS over time, where the RCS means of the DAA curve is −11.45 dBm^2^, and that of the other set of data is −10.72 dBm^2^. These results show that DAA is feasible and accurate to analyze the dynamic RCS of the target.

## 3. Model of Tandem Helicopter

**Figure 3:**
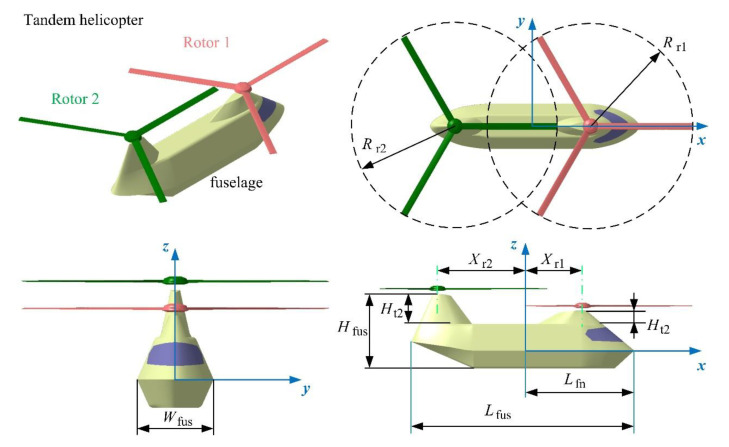
Tandem helicopter model and its size distribution.

With reference to the outline layout of the CH-46 Sea Knight helicopter and the advanced design of the RAH-66 Comanche helicopter [5,8], the model of the tandem helicopter is established as shown in Figure 3, where *L*_fus_, *W*_fus_ and *H*_fus_ are the lengths, width and height of the helicopter fuselage, respectively. *L*_fn_ is the distance from the vertex of the nose to the *yz* plane. *R*_r1_ and *R*_r2_ are the radius of rotor 1 and 2, respectively. When the rotor is not tilted, the rotor axis is parallel to the *z*-axis, and *X*_ri_ (*i* = 1 or 2) is the distance from the axis of rotor *i* to the *z*-axis. *H*_t1_ and *H*_t2_ are the height of the rotor 1 tower and rotor 2 towers, respectively, where the sizes of these dimensions are presented in Table 1.

**Figure 4:**
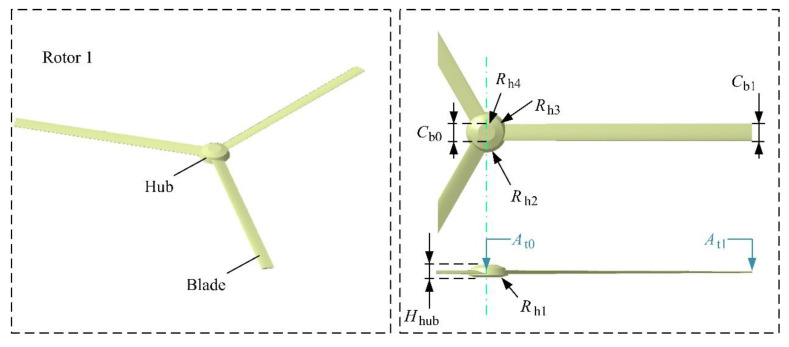
Model and size distribution of rotor 1.

The model of rotor 1 is shown in Figure 4, where *C*_b0_ and *C*_b1_ represent the chord length at the root and the tip of the blade, respectively. *H*_hub_ is the height of the rotor hub, and its value is given in Table 2. *A*_t0_ and *A*_t1,_ respectively, represent the torsion angle of the root and the tip of the blade. *R*_h1_~*R*_h4_ are the radius of each circular edge of the hub from bottom to top in turn. The model of Rotor 2 is the same in size as Rotor 1, and it can be regarded as the result of the symmetry of Rotor 1 about the *yz* plane and then translation.

**Figure 5:**
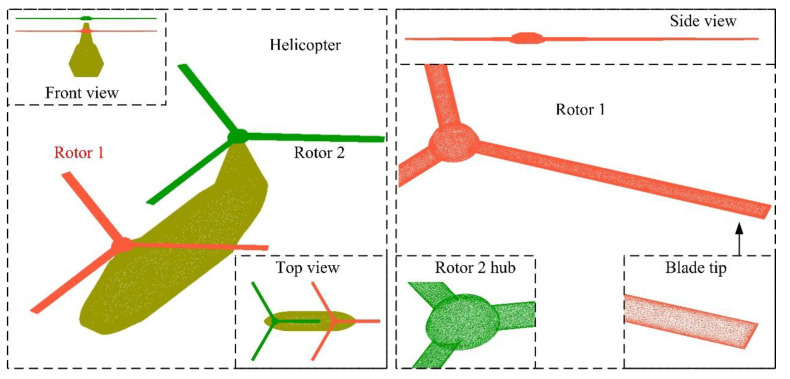
High-precision mesh on the surface of the helicopter model.

High-precision unstructured grid technology is used to divide the surface of the helicopter model, as shown in Figure 5, where dense mesh processing is used to handle edges and surfaces with large curvature changes. For the rotor, the leading edge, trailing edge, blade tip and hub edge have smaller dimensions and obvious split angle. For the surface of the helicopter fuselage, the geometrical dimensions of the sidelines, the nose, the top of the rotor tower and the edge of the tail are small.

## 4. Results and Discussion

**Figure 6:**
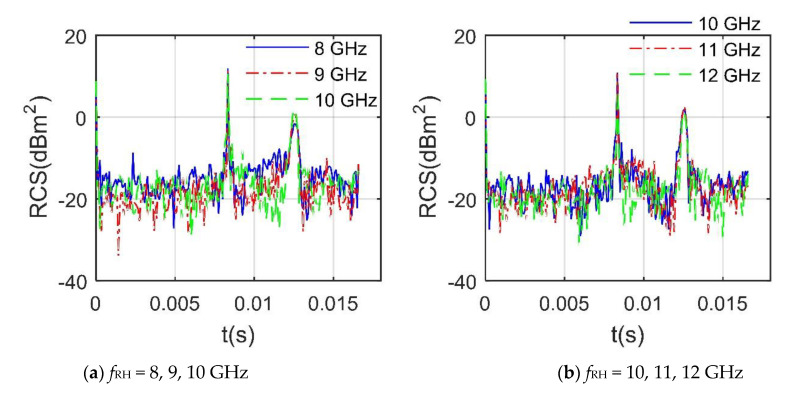
Dynamic RCS of rotor 1 at different *f*_RH_, *α* = 30°, *β* = 5°, *n*_r1_ = 1200 r/min.

Figure 6 presents that the RCS curves of the rotor under various *f*_RH_ in the X-band are obviously different, including fluctuation range, peak size and curve shape, while their peak positions are relatively consistent. The 5 RCS curves have peaks exceeding −1.651 dBm^2^ at *t* = 0, 8.33 × 10^−3^, and 1.26 × 10^−2^ s. For the first RCS peak, the trailing edge of a blade is perpendicular to the incident radar wave, which makes a large number of strong scattering sources appear on the surface near the trailing edge. At *t* = 8.33 × 10^−3^ s, the peak at 8 GHz is 11.88 dBm^2^, while that at 12 GHz is 9.763 dBm^2^, because the rotor rotates 60°, making the leading edge of a blade perpendicular to the radar wave, which contributes to a significant increase in RCS. The RCS means of rotor 1 at different *f*_RH_ is shown in Table 3, where the maximum difference between these means reached 0.952 dBm^2^. In order to capture the radar characteristics of the tandem helicopter, DAA is used to analyze its dynamic RCS under more influential factors in the following content.

### 4.1. Influence of Individual Rotor

**Figure 7:**
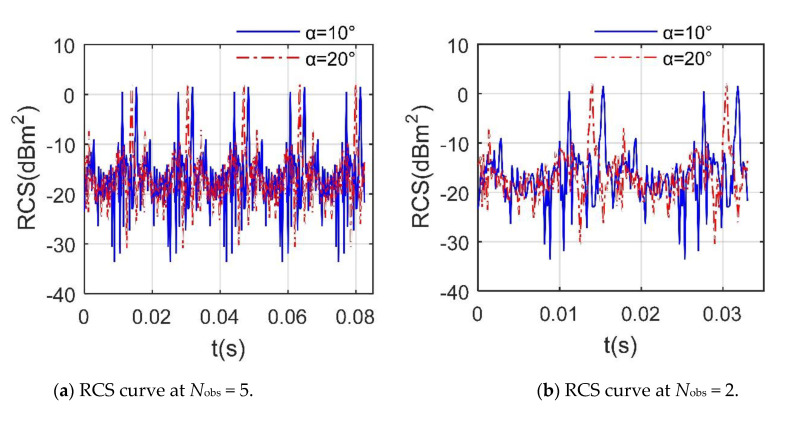
Periodic characteristics of dynamic RCS of rotor 1, *f*_RH_ = 10 GHz, *β* = 5°, *n*_r1_ = 1200 r/min.

Figure 7 provides that the RCS of rotor 1 under a given azimuth angle shows obvious periodic characteristics, where the inclination of the rotor disk and the attitude angle of the fuselage are both equal to 0. The minimum period of the dynamic RCS of the rotor at this time is equal to 0.0165 s, which is exactly equal to the basic passage time, where *N*_obs_ is a multiple of the basic passage time. Although the RCS curves under these 2 azimuths have many differences, including peak, fluctuation range and shape, their minimum periods are the same, where the peak of the RCS curve with *α* = 10° is 1.538 dBm^2^, and that at *α* = 20° is 2.009 dBm^2^, because for a single rotor with a fixed attitude, different observation angles determine the starting point of the rotor dynamic RCS, and the continuous rotation of the rotor will cause its blades to enter the next similar process after the basic passage time. This shows that DAA can well capture the periodic characteristics of the rotor RCS.

**Figure 8:**
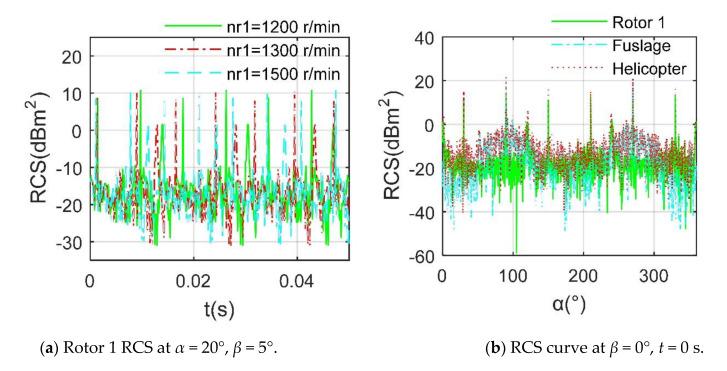
Electromagnetic scattering characteristics of rotor 1, *f*_RH_ = 10 GHz.

Figure 8 indicates that as the rotation speed increases, the RCS period of the rotor is obviously shortened. When *n*_r1_ = 1300 r/min, the dynamic RCS period of rotor 1 is equal to 0.0153 s, while this value is only 0.0132 s at 1500 r/min because the greater rotation speed allows any blade of the rotor to quickly complete the movement within the basic passage time. For the RCS results compared to fuselage and helicopter, it can be observed that the overall RCS level of the rotor is the lowest among the three, where the RCS means of the rotor is equal to −9.0752 dBm^2^, that of the fuselage is −7.3684 dBm^2^, and that of the helicopter is −3.0947 dBm^2^ because the overall configuration of the rotor is very thin, its projection in the radar incident direction is much smaller than that of the fuselage and helicopter. In addition, despite the huge size of the fuselage, it uses a better shape design, including inclined side panels, flat nose and sharp rotor tower. The rotor has a similar RCS order of magnitude compared with the fuselage and contributes a lot to the main peak of the helicopter, while the influence of rotation speed on rotor RCS is mainly reflected in the period.

### 4.2. Influence of Observation Angle

**Figure 9:**
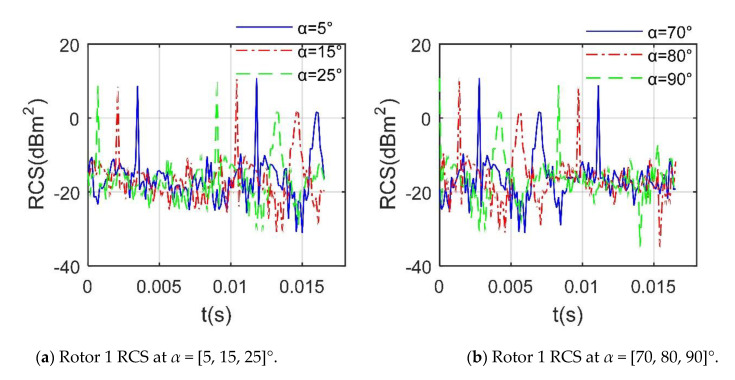
Dynamic RCS of rotor 1 at different azimuths, *f*_RH_ = 10 GHz, *β* = 5°, *n*_r1_ = 1200 r/min.

Figure 9 reveals that at a given *β*, the influence of the azimuth on the dynamic RCS of rotor 1 is mainly reflected in the initial RCS, where the inclination angle of the rotor disc and the attitude angle of the fuselage are both equal to 0. The RCS of the rotor at different azimuth angles has great similarities in other aspects, including peak size, curve shape and variation range. It can be found that the maximum peak of these RCS curves is equal to 10.86 dBm^2^, and the second-largest peak is 8.754 dBm^2^. The different starting points of these RCS curves are mainly due to the difference in the angle between the rotor and the incident radar wave at the initial moment, resulting in a difference in the comprehensive effect of the blade on the deflection of the radar wave. The similarity of these RCS curves is mainly because the rotor is in a horizontal position, and the elevation angle is small. In addition to the initial design of the propeller hub, these together lead to the similarity of the dynamic RCS of the rotor at different azimuths. These results indicate that the influence of the azimuth angle on the dynamic RCS of the horizontally placed rotor is mainly reflected in the initial RCS.

**Figure 10:**
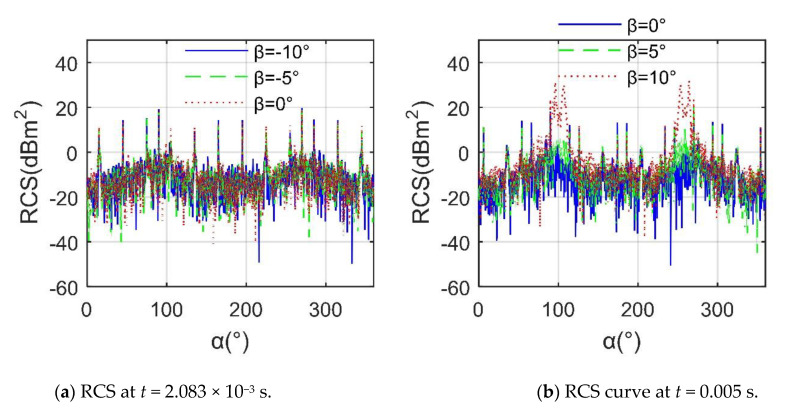
RCS of helicopter at various *β*, *f*_RH_ = 10 GHz, *n*_r1_= *n*_r2_ = 1200 r/min, *A*_df_ = *A*_dr_ = 0°, *γ* = *θ* = 0°.

Figure 10 manifests that a positive *β* has a significant impact on the helicopter’s RCS~*α* at a given moment. For *t* = 2.083 × 10^−3^ s, the performance of the RCS curves is very similar, including peaks, curve trends and fluctuations, where the average value of the RCS curve at *β* =–10° is −3.8226 dBm^2^, that at *β* = −5° is −3.5141 dBm^2^, and that at *β* = 0° is −3.5755 dBm^2^. At this time, as *β* decreases from 0, the strong scattering source of the helicopter is mainly concentrated on the curved surface at the bottom of the fuselage; thus, these three RCS curves are relatively close. For *t* = 0.005 s, the three RCS curves are quite different, including peak size, fluctuation range, peak position and curve shape, where the peak of the curve at *β* = 0° is 18.5 dBm^2^ at *α* = 270°, that at *β* = 5° is 21.14 dBm^2^ at *α* = 270°, and that at *β* = 10° is 31.95 dBm^2^ at *α* = 264°. In addition, the average of the RCS curve at *β* = 0° is −3.6538 dBm^2^, and the peak value of the RCS curve at *β* = 10° is 14.4624 dBm^2^. The reason for the significant increase in RCS here is that the surface of the nose, the sides of the fuselage, the rotor towers, the front and rear edges of the blades, and the tip of the blade have become new scattering sources. These results indicate that although this helicopter uses a preliminary tilt design, it still has unfavorable RCS performance at a larger elevation angle.

### 4.3. Influence of Rotor Disk Inclination

**Figure 11:**
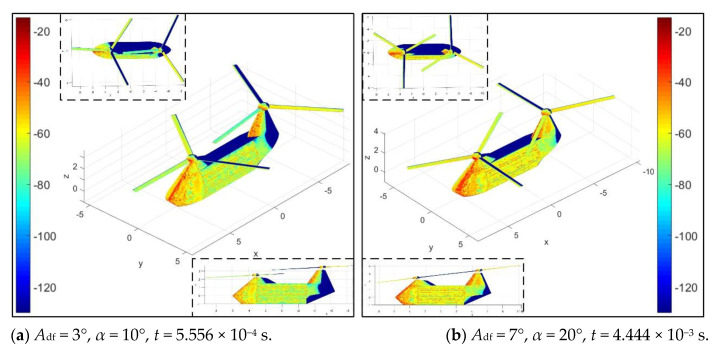
Surface scattering characteristics of helicopter at various *A*_df_, *f*_RH_ = 10 GHz, *n*_r1_= *n*_r2_ = 1200 r/min, *β* = 0°, *A*_dr_ = 0°, *γ* = *θ* = 0°, RCS unit: dBm^2^.

Figure 11 investigates that *A*_df_ mainly affects the distribution of scattering sources on the rotor surface under current conditions. For the case of *A*_df_ = 3°, at this time, the rotor has rotated 4°, the surface of the blades is green and yellow on the whole, and a small amount of red and yellow are distributed on the hub. When *A*_df_ is increased to 7°, darker red and orange-red appear in the illumination area of the propeller hub; because the propeller hub is designed as a rotating body, the influence of the azimuth angle and the rotation angle of the rotor on its RCS is negligible, while the change in the intensity of the scattering source on the surface of the hub is determined by the inclination of the rotor disc. Coupled with the influence of the upper position angle and the rotation of the rotor, the orange-red on the surface of the blade is obviously increased, and the green is greatly reduced, where the rotor has rotated 32°. As the azimuth angle increases from 10° to 20°, the red area of the nose and rotor tower base increases greatly, and the original main body green on the side of the fuselage is also replaced by orange and yellow. These results show that DAA can well describe the variation of scattering sources on the helicopter surface under different azimuth and rotor disc inclination.

**Figure 12:**
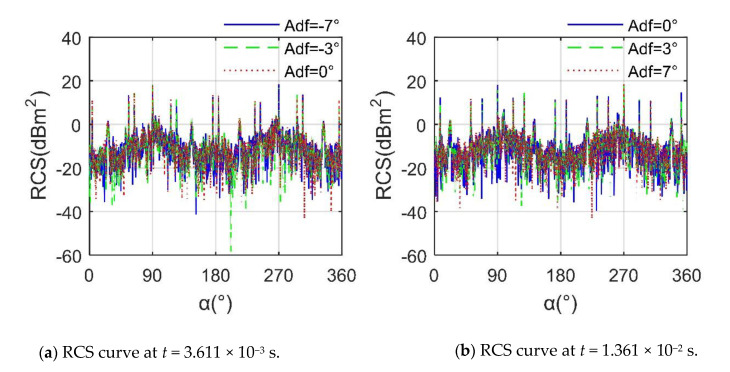
RCS of helicopter at various *A*_dr_, *f*_RH_ = 10 GHz, *n*_r1_= *n*_r2_ = 1200 r/min, *β* = 0°, *A*_dr_ = 0°, *γ* = *θ* = 0°.

Figure 12 shows that the impact of different *A*_df_ values on helicopter RCS is mainly reflected in small peaks and local fluctuations and has little impact on large peaks and overall trends. For the curve at *t* = 3.611 × 10^−3^ s, the average of the RCS at *A*_df_ = −7° is −4.4673 dBm^2^, that at *A*_df_ = −3° is −3.8614 dBm^2^, and that at *A*_df_ = 0° is–3.6651 dBm^2^ because as *A*_df_ increases from −7° to 0°, the influence of the tip surface of the propeller and the front and rear edges of the propeller on the RCS indicator gradually increases. At *α* = 145.5°, the peak of the RCS curve at *A*_df_ = −7° is 0.7592 dBm^2^, while that at *A*_df_ = −3° is 3.445 dBm^2^; this is also due to the contribution of the scattering source on the surface of the blade. For the case of *t* = 1.361 × 10^−2^ s, the RCS means of *A*_df_ = 0° is −3.7076 dBm^2^, that of *A*_df_ = 3° is −3.8724 dBm^2^, and that of *A*_df_ = 7° is −4.3679 dBm^2^, because, with the increase of *A*_df_ from 0° to 7°, the strong scattering source of the propeller tip is continuously weakened. Although the propeller hub brings adverse effects, the entire rotor disc is beneficial to deflect the radar waves. These results show that the influence of the forward tilt of the main rotor on the helicopter RCS cannot be ignored.

**Figure 13:**
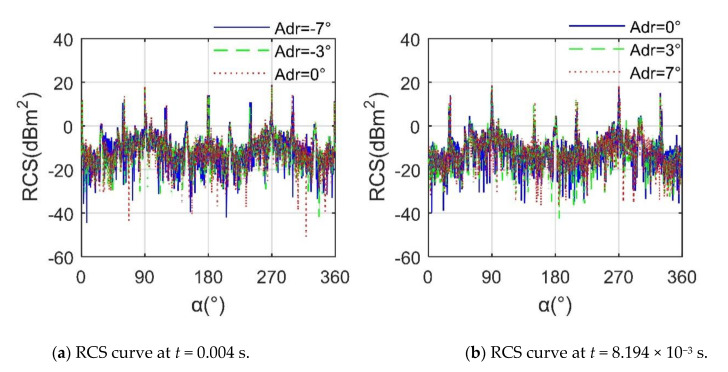
RCS of helicopter at various *A*_dr_, *f*_RH_ = 10 GHz, *n*_r1_= *n*_r2_ = 1200 r/min, *β* = 0°, *A*_df_ = 5°, *γ* = *θ* = 0°.

Figure 13 presents that the influence of different *A*_dr_ on helicopter RCS is mainly reflected in small peaks and fluctuations in lateral azimuth. When *t* = 0.004 s, in the azimuth range of 37.75°~105.3° and 258°~309.5°, the three curves have an obvious difference, where the average RCS at *A*_dr_ = −7° is −3.9581 dBm^2^, that at *A*_dr_ = −3° is −3.9348 dBm^2^, and that at *A*_dr_ = 0° is −3.8972 dBm^2^, because at this time the projection area of the rotor disk in the lateral direction is significantly increased, and then a larger illumination area and more scattering sources appear. For *t* = 8.194 × 10^−3^ s, the mean of the RCS curve at *A*_dr_ = 0° is −4.2673 dBm^2^, that at *A*_dr_ = 3° is −3.8744 dBm^2^, and that at *A*_dr_ = 7° is −4.2225 dBm^2^, where within the range of 282.15°~315.5° azimuth, the three RCS curves are quite different, because the rotor is tilted to the negative direction of the *y*-axis, although the scattering characteristics of the blade tip are improved, the RCS performance of the hub in this lateral direction is higher. These results show that DAA can well capture the effect of rotor disk tilt on the helicopter. Although the rotor adopts a preliminary stealth design, the effect of the rotor disk tilt on the helicopter RCS is also obvious.

### 4.4. Influence of Attitude Angle

**Figure 14:**
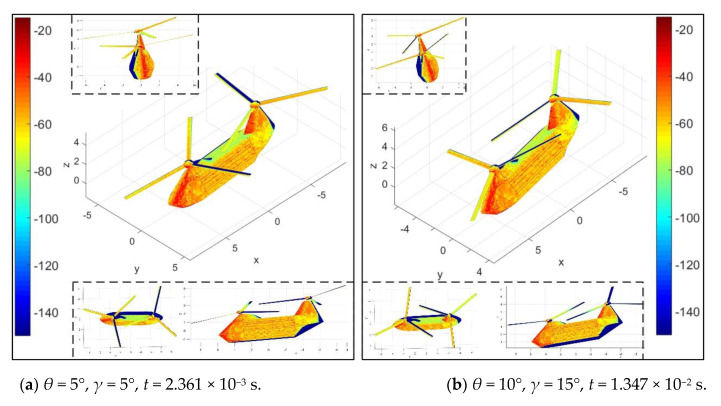
Surface scattering characteristics of helicopter at various attitude angles, *f*_RH_ = 10 GHz, *n*_r1_= *n*_r2_ = 1200 r/min, *α* = 25°, *β* = 0°, *A*_df_ = *A*_dr_ = 5°, RCS unit: dBm^2^.

Figure 14 shows the attitude angle has a great influence on the electromagnetic scattering characteristics of the helicopter surface under current conditions, which mainly reflects on the nose, rotor tower and blades. For *θ* = *γ* = 5° and *t* = 2.361 × 10^−3^ s, the rotor has rotated 17°, where the strong scattering sources are mainly concentrated in the front of the nose, the front edge of the front tower, the front side of the rear tower and the front side of the hub. The side of the fuselage is orange and yellow, and orange and green are present on the surface of the blade. When *θ* = 10°, *γ* = 15° and *t* = 1.347 × 10^−2^ s, the attitude angle of the fuselage has been increased, and the rotor has rotated 97°, the red color of the front hub decreases because of the occlusion of the blade roots, the red color of the rear hub increases slightly, the light yellow on the top of the fuselage increases sporadically, and the green at the rear of the fuselage has a small area increase. The positive increase of the pitch angle is conducive to the reduction of the RCS of the lower surface of the nose, but it is not good for the RCS level of the upper surface of the nose. For the roll angle, the positive increase is beneficial to the partial deflection of the radar wave on the side of the fuselage at this time, while it is not welcome to the top and the lower half of the side of the fuselage. These results show that DAA can well describe the transfer of scattering source distribution on the fuselage surface when the attitude angle changes.

**Figure 15:**
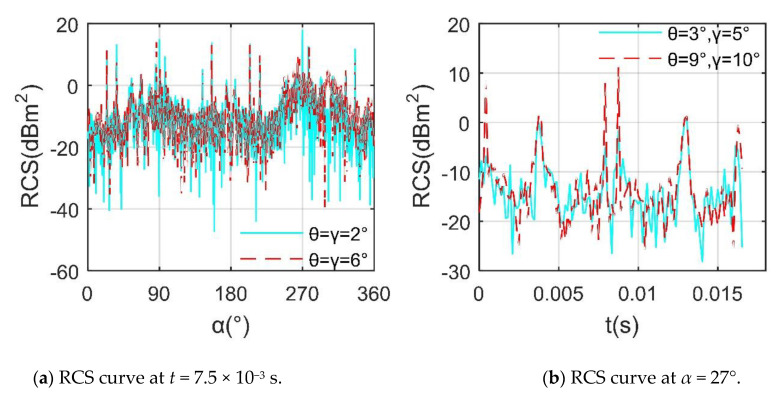
RCS of the helicopter at various attitude angles, *f*_RH_ = 10 GHz, *n*_r1_= *n*_r2_ = 1200 r/min, *β* = 0°, *A*_df_ = *A*_dr_ = 7°, RCS unit: dBm^2^.

Figure 15 provides that there are obvious differences in the RCS~*α* and RCS~*t* curves of helicopters at different attitude angles. For *t* = 7.5 × 10^−3^ s, the overall trend and fluctuation range of the two RCS curves are similar, while the average value and peak value are quite different, where the RCS means of the curve under *θ* = *γ* = 2° is −4.4013 dBm^2^, and that under *θ* = *γ* = 6° is −3.8202 dBm^2^, the main reason for the increase in the RCS index is the contribution of the large-area strong scattering source on the curved surface near the nose. There are 8 peaks exceeding 10 dBm^2^ on the *θ* = *γ* = 2° curve, and this indicator is 6 on the *θ* = *γ* = 6° curve, where the maximum value of the former curve is 17.99 dBm^2^ at *α* = 270°, while that of the latter is 15.57 dBm^2^ at *α* = 86.25°. For *α* = 27°, the main difference between the two curves is reflected in the peak size and fluctuation range, where the RCS means of the curve at *θ* = 3° and *γ* = 5° is −10.1944 dBm^2^, that of the other is −4.6993 dBm^2^, the main factors for the increase of this RCS index include the enhancement of scattering sources on the lower part of the side of the fuselage, the top of the fuselage, the leading edge of the rotor tower, and the hub. These results indicate that the helicopter’s RCS is unpopular at a larger attitude angle, and the fluctuation range of its dynamic RCS is also undesirable.

### 4.5. Doppler Feature Analysis

**Figure 16:**
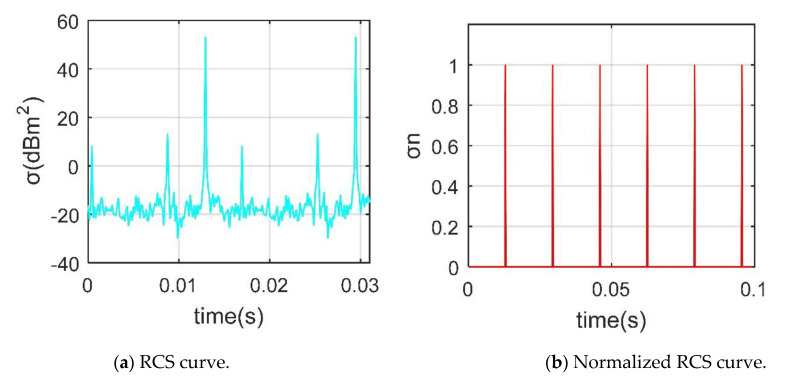
RCS of the rotor 1, *α* = 27°, *β* = 0°, *A*_df_ = *A*_dr_ = 0°, *f*_RH_ = 10 GHz, *V*_m_ = 0 m/s, *n*_r1_= 1200 r/min.

**Figure 17:**
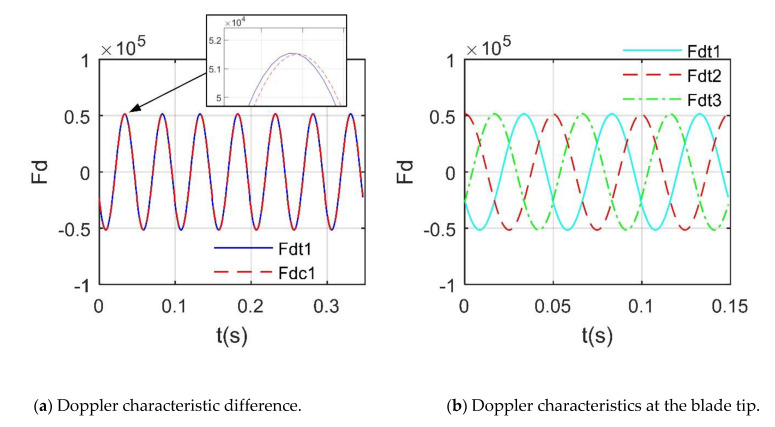
Doppler feature of the rotor 1, *α* = 27°, *β* = 0°, *A*_df_ = *A*_dr_ = 0°, *f*_RH_ = 10 GHz, *V*_m_ = 0 m/s, *n*_r1_= 1200 r/min.

Figure 16 shows that the normalized RCS compresses most of the original smaller values [10,27]. For the rotor RCS, the maximum reaches 53.34 dBm^2^, where the smaller peaks are 13.36 dBm^2^ and 8.27 dBm^2^. After normalization, only 6 new peaks appeared within 0~0.1 s, and the original small peaks were submerged because the maximum and minimum values of RCS are too different. Figure 17 provides that the maximum value of *F*_dt_ is 515.493 × 10^2^ Hz, slightly larger than *f*_dmax_ (515.221 × 10^2^ Hz), where *F*_dt1_ is the Doppler frequency at an intersection point (recorded as *P*_1_) of the first blade tip and the leading edge, *F*_dc1_ is the Doppler frequency at the center of the first blade tip, the first blade is the blade along the *x*-axis in the initial state [28,29]. This rotor 1 is composed of 3 blades, the tip of each blade will produce the most significant Doppler feature, and the extraction of other *F*_dt_ information is shown in Figure 17b. It can be found that in a minimum period, there are 3 maximums and 3 minimums, which represents the alternating Doppler frequency of the 3 blade tips during the rotation. These results show that it is feasible and accurate for DAA to analyze the Doppler characteristics of the rotor.

## 5. Conclusions

Based on the presented dynamic analysis approach, the factors affecting the radar cross-section in the X-band of the tandem helicopter are studied and discussed in detail. Through these investigations and analyses, the following conclusions can be drawn:

(1) In the current band, the dynamic RCS average index of the helicopter under a given heading azimuth angle shows a decrease-increase-decrease change with the increase of the radar wave frequency;

(2) The RCS of the rotating rotor shows strong dynamic characteristics and obvious periodic characteristics under a given head azimuth angle while increasing the rotation speed can reduce this dynamic period;

(3) The RCS of the rotor and the helicopter under different observation angles are obviously different. The positive increase of the elevation angle has a greater impact on the peak and the average value of the helicopter’s RCS;

(4) The inclination angle of the rotor disc mainly affects the small peak value of the helicopter RCS. A larger attitude angle will adversely affect the peak, maximum value and dynamic characteristics of the helicopter RCS.

## Figures and Tables

**Table 1:** The main dimensions of the helicopter model.

Parameter	*L*_fus_	*W*_fus_	*H*_fus_	*L*_fn_	*R*_r1_
Value (m)	12.5	2.7	4.163	6	6.15
Parameter	*R*_r2_	*H*_t1_	*H*_t2_	*X*_r1_	*X*_r2_
Value (m)	6.15	0.761	1.663	3.1	5.02

**Table 2:** Main dimensions of rotor 1.

Parameter	Airfoil	*C*_b0_ (m)	*A*_t0_ (°)	*A*_t1_ (°)	*C*_b1_ (m)
Value	TSAGI 8%	0.4	12	3	0.4
Parameter	*R*_h1_	*R*_h2_	*R*_h3_	*R*_h4_	*H*_hub_
Value (m)	0.293	0.47	0.39	0.2	0.29

**Table 3:** Dynamic RCS mean of rotor 1 at different *f*_RH_, *α* = 30°, *β* = 5°, *n*_r1_ = 1200 r/min.

*f*_R_/GHz	8	9	10	11	12
Mean RCS (dBm^2^)	−7.8077	−8.1684	−7.9870	−8.1797	−8.7597

## Data Availability

Not applicable.
